# Antitumor activity of RUNX3: Upregulation of E-cadherin and downregulation of the epithelial–mesenchymal transition in clear-cell renal cell carcinoma

**DOI:** 10.1515/biol-2022-0494

**Published:** 2022-12-02

**Authors:** Ruo-Nan Yang, Fu-Rong Zhou, Hong-Yang Wang, Qing-Hai Wang, Jian-Lei Ji, Tao Huang, Chen Guo, Zhen Dong, Yan-Wei Cao

**Affiliations:** Department of Renal Transplantation and Urology, The Affiliated Hospital of Qingdao University, No. 59 Haier Road, Qingdao, Shandong, China; Department of Pharmacy, Yantai Yuhuangding Hospital, Yantai, Shandong, China

**Keywords:** E-cadherin, enEMT, clear-cell renal cell carcinoma, *RUNX3*

## Abstract

RUNX3 is a transcription factor and tumor suppressor that is silenced or inactivated in diverse tumors. The effect of RUNX3 on the epithelial–mesenchymal transition in clear-cell renal cell carcinoma (CCRCC) remains unclear. We determined the expression of *RUNX3* and E-cadherin in tumor tissues and adjacent normal tissues of 30 CCRCC patients; established cultured CCRCC cells with the overexpression of *RUNX3*; and examined the *in vivo* tumorigenic function of *RUNX3* in a nude mouse xenograft model of CCRCC. *RUNX3* and E-cadherin were downregulated in human CCRCC samples. Cell lines with *RUNX3* overexpression had reduced cell proliferation, invasion, and migration, a prolonged cell cycle, increased apoptosis, and increased expression of E-cadherin. In the nude mouse xenograft model of CCRCC, tumors with the overexpression of *RUNX3* had smaller volumes and weights and had increased expression of E-cadherin. In conclusion, *RUNX3* overexpression increased the level of E-cadherin and inhibited the proliferation, invasion, and migration of CCRCC *in vitro* and *in vivo*. *RUNX3* has potential use as a biomarker for prognostic monitoring of CCRCC and as a therapeutic target for the treatment of this cancer.

## 1 Introduction

Renal cell carcinoma (RCC) originates from the parenchymal cells of the renal tubular epithelium and is the most common malignant tumor of the kidney. Globally, RCC accounts for 2.4% of all cancers and 1.7% of all cancer-related deaths [1]. Clear-cell RCC (CCRCC) is the most common histological type of RCC in adults, and it accounts for 90% of all RCCs [2]. A recent study of the incidences of different cancers reported that CCRCC had the sixth highest incidence among men and the tenth highest among women, accounting for 5 and 3% of all tumor diagnoses, respectively [3,4]. The incidence of CCRCC has continued to increase over time. This cancer is generally not sensitive to chemotherapy or radiotherapy, and surgery is the main treatment. Patients with early-stage CCRCC often lack specific clinical manifestations, and 20–30% of these patients receive diagnoses only after the development of local or distant metastasis. However, surgery is usually unsuitable for patients with advanced CCRCC, and there is therefore an urgent need for new treatments for these patients. To improve the early diagnosis and treatment of CCRCC, it is necessary to analyze the mechanisms of its onset and progression to identify new biomarkers and therapeutic targets.

Proteins in the runt-related transcription factor family – *RUNX1, RUNX2*, and *RUNX3* – play important roles in the transforming growth factor-β (TGF-β) signaling pathway [4]. Early studies concluded that *RUNX3* functioned as a tumor suppressor and is located on human chromosome 1 at 1p36.11, a region that is missing in many types of cancers [5]. This led to speculation that this region contains an important tumor suppressor gene. Recent studies examined the role of *RUNX3* in tumor suppression and the prevention of metastasis in cervical cancer [6], endometrial cancer [7], and gastric cancer [8]. A 2002 study reported that *RUNX3* functioned as a tumor suppressor in gastric epithelial cells and that this gene was inactivated in up to 80% of primary gastric tumors [9]. Other research reported the presence of gastric hyperplasia and tumors in *RUNX3*-knockout mice, and this was related to decreased apoptosis and sensitivity to TGF-β [4,10]. Although many studies showed that *RUNX3* can inhibit cell migration, invasion, and apoptosis, a few studies examined its function in the onset and progression of CCRCC.

The epithelial–mesenchymal transition (EMT) is a physiological process in which epithelial cells lose their polarity and develop the characteristics of stromal cells. Researchers first recognized the EMT as a normal physiological process during embryonic growth [11]. During this process, epithelial cells become immature and can no longer differentiate, and they lose their cell adhesion properties, leading to increased plasticity, invasiveness, motility, and anti-apoptotic activities. Researchers now recognize the EMT as an essential event in the pathogenesis of many cancers and other diseases [12]. During tumor formation, increased EMT activity is contributed to tumor invasion, metastasis, and development of drug resistance. There is now evidence that the EMT plays an important role in the occurrence and development of liver cancer [13], melanoma [14], colorectal cancer [15], breast cancer [16], and other cancers. Downregulation of epithelial cadherin (E-cadherin) is associated with decreased cell adhesion, an important event in the EMT that is necessary for tumor invasion and metastasis.

In the present study, we examined the function of *RUNX3* as a tumor suppressor gene in CCRCC. In particular, we examined the effect of *RUNX3* overexpression on apoptosis and inhibition of tumor migration and invasion. Our general aim was to examine the use of *RUNX3* as a prognostic indicator of CCRCC and its potential use as a target for the treatment of this cancer.

## 2 Materials and methods

### 2.1 Tissue collection and immunohistochemistry

Thirty human specimens of CCRCC and adjacent non-cancerous tissues were collected from the pathology department of our hospital. Immunohistochemistry was performed using the Histostain-Plus SP kit (Bioss Antibodies, Beijing, China) according to the manufacturer’s instructions.


**Informed consent**: Informed consent has been obtained from all individuals included in this study.
**Ethical approval**: The research related to human use has been complied with all the relevant national regulations, institutional policies and in accordance with the tenets of the Helsinki Declaration, and has been approved by the Ethics Committee of the Affiliated Hospital of Qingdao University.

### 2.2 Cell culture and transfection

Two cell lines of human CCRCC (ACHN and 786-O) were obtained from Genechem (Shanghai, China) and cultured in the Central Laboratory of the Affiliated Hospital of Qingdao University. The 786-O cells were cultured in RPMI1640 (Gibco, USA) supplemented with 10% fetal bovine serum (FBS) (Ausbian, Australia). The ACHN cells were cultured in dulbecco’s modified eagle medium (Corning, USA) supplemented with 10% FBS (Ausbian, Australia).

Lentivirus LV-RUNX3 (21371-1) with a green fluorescent protein (*GFP*) tag were used to transfect cells and construct *RUNX3*-overexpressing cells (OE cells). Lentivirus CON220 (negative control) with a *GFP* tag was used to establish normal control (NC) cells. Both of these lentiviruses were obtained from Genechem (Shanghai, China). At 18 h after transfection, the medium was replaced with complete medium. At 72 h, the transfection was complete and cells were viewed under a fluorescence microscope.

### 2.3 Western blotting

Proteins were extracted into a buffer with detergent, separated by electrophoresis in sodium dodecyl sulfate–polyacrylamide gels, and then blotted onto nitrocellulose membranes. The membranes were incubated at 4°C with antibodies against RUNX3 (ab135248, 1:1,000 dilution, Abcam) and GAPDH (sc-32233, 1:1,000 dilution, Santa Cruz Biotechnology). After washing, the membranes were incubated with a secondary Mouse IgG antibody (sc-2005, 1:2,000 dilution; Santa Cruz Biotechnology) for 2 h at room temperature. Blots were visualized using electrochemiluminescence (Amersham Pharmacia Biotech). The molecular weights of RUNX3 and E-cadherin were 44 and 120 kDa, respectively.

### 2.4 Clone formation and MTT assays

The cell suspension was diluted and different numbers of cells were placed into prepared dishes (50, 100, 200, and 500 cells per dish). Then, the cells were placed in a humidified incubator at 37°C. Cloning was terminated when cell clumps were observable after 2–3 weeks. Then, the cells were fixed with paraformaldehyde for 15 min, stained with crystal violet for 30 min, and then washed and stained.

Before culture termination, 20 μL of 5 mg/mL MTT (Sigma, USA) was added for 4 h, and the cells were then lysed with 100 μL dimethylsulfoxide to dissolve the formazan crystals [17,18,19]. The sample was oscillated for 2–5 min, and the absorption was then measured at 490 nm. The number of living cells was measured after 1, 2, 3, 4, and 5 days.

### 2.5 Transwell and cell scratch assays

For cell migration experiments, growth medium was added to a 24-well plate, and a cell suspension was added to the upper chamber of the Transwell. After 24 h, the medium was removed, fixed with paraformaldehyde for 10 min, stained with crystal violet for 20 min, rinsed with PBS, and then photographed for observation.

For cell invasion experiments, matrix gelatin was dripped into the upper chamber of the Transwell. After standing for 1 h, it was transferred into a 24-well plate. The rest of the procedures were the same as in the cell migration experiments.

For the cell scratch assay, a cell suspension was added dropwise into a 6-well plate and incubated at constant temperature and humidity. When the cell density was 90%, a scratch mark was made using a pipette tip that was perpendicular to the previous marking line. The previously streaked cells were then washed with PBS, and images were recorded at 0, 12, and 24 h.

### 2.6 RNA extraction and RT-PCR

Total RNA was extracted from cells and enriched using the TRIzol miRVana isolation protocol (Invitrogen, USA). When the RNA precipitate was transparent, RNase-free water was added until it was completely dissolved, and the concentration and quality of the extracted RNA were determined using a Nanodrop 2000C spectrophotometer. A Promega M-MLV kit was used for the reverse transcription of total RNA into cDNAs. The mRNA levels were evaluated using qRT-PCR with following primers: *GAPDH*-forward: 5′-TGACTTCAACAGCGACACCCA-3′, *GAPDH*-reverse: 5′-CACCCTGTTGCTGTAGCCAAA-3′; *RUNX3*-forward: 5′-GCCTTCAAGGTGGTGGCATT-3′, *RUNX3*-reverse: 5′-TCAGCGGAGTAGTTCTCGTCATT-3′.

### 2.7 Flow cytometry

The cell cycle assay was performed using propidium iodide fluorescence-activated cell sorting (PI-FACS). After the cell suspension was prepared, the cells were washed with D-Hanks solution, centrifuged, and fixed with 75% alcohol. The fixative was removed, the cells were washed again with D-Hanks, a cell staining solution was added, and cells were then detected using flow cytometry. The cell staining solution consisted of 40× PI (2 mg/mL; Sigma, YSA):100× RNase (10 mg/mL; Fermentas, China):1× D-Hanks = 25:10:1,000.

Apoptosis was detected by Annexin V-APC staining with flow cytometry using an apoptosis detection kit (Bestbio, China) following the manufacturer’s instructions.

### 2.8 Tumorigenicity

The tumorigenicity of 4-week-old female BALB/c nude mice (GemPharmatech, Jiangsu, China) was determined as described previously [20]. Sixteen mice were randomly assigned into two groups (8 per group), and the animals then received subcutaneous injections of ACHN cells that were transfected with the *RUNX3* OE vector or the NC vector. After 14 days, tumors at the injection site were palpated, and tumors were visually scored weekly when they were observable. Tumor size and weight were determined by measuring excised tumors after euthanasia, at 56 days after cell injection. These experiments were performed according to the guidelines of the Animal Care and Use Committee of the National Institutes of Health and were approved by the Ethics Committee of the Affiliated Hospital of Qingdao University.

### 2.9 Statistical methods

GraphPad Prism version 9.0, ImageJ-FIJI, and SPSS version 24 were used for statistical analysis. Student’s *t*-test was used for comparisons when variables had normal distributions, and the Mann–Whitney test was used when variables had non-normal distributions. A *P*-value of below 0.05 indicated statistical significance. Each measurement was performed three times, and average values and standard deviation were presented.

## 3 Results

### 3.1 Human CCRCCs have downregulation of RUNX3 and E-cadherin and upregulation of EZH2

**Figure 1:**
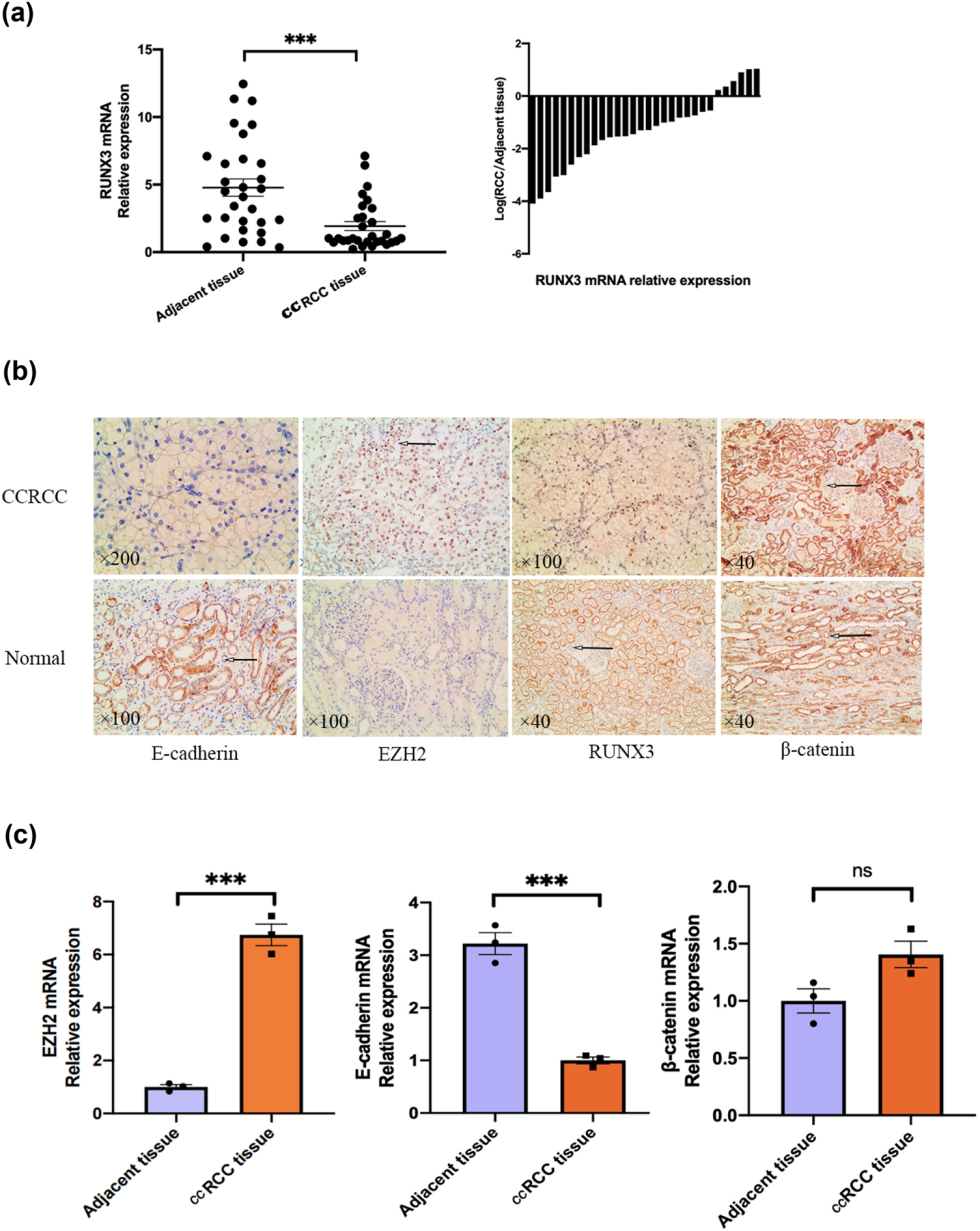
Human CCRCCs have downregulation of *RUNX3* and E-cadherin and upregulation of *EZH2*. (a) *RUNX3* expression in CCRCC tissues and adjacent normal tissues (RT-PCR). (b) Expression of marker proteins in CCRCC tissues and adjacent normal tissues (immunohistochemistry, scale bars on the bottom left of each image). (c) Expression of marker mRNAs in CCRCC tissues and adjacent normal tissues (RT-PCR). Each assay was performed at least three times, and values are means ± standard errors of the means (SEMs), ***P < 0.001, ns *P* > 0.05.

We examined the carcinoma tissues and adjacent normal tissues of 30 patients with CCRCC (Table 1). There were 17 males and 13 females, the average age was 54.8 ± 2.4 years old, and the mean tumor diameter was 6.5 ± 2.3 cm. Measurements of the expression of *RUNX3* mRNA in these patients indicated a significantly lower level in cancer tissues than in adjacent tissues (Figure 1a). Because of the small sample size, we could not draw meaningful conclusions regarding the relationship of *RUNX3* expression with tumor stage. Then, we performed the immunostaining of these samples for four proteins that have established roles in carcinogenesis (Figure 1b and Table 1). The expression of RUNX3 and E-cadherin was lower in CCRCC tissues, the expression of EZH2 was higher in CCRCC tissues, but the expression of β-catenin was similar in CCRCC and normal tissues. RT-PCR of these tissues showed that *EZH2* had greater expression in tumor tissues, E-cadherin had decreased expression in tumor tissues, and β-catenin had similar expression in tumor and normal tissues (Figure 1c)

**Table 1:** Characteristics and outcomes of CCRCC tissue and adjacent normal tissue

Clinical feature	RCC tissue (*n* = 30)	Adjacent tissue (*n* = 30)	*P*
Characteristics			
Age (years)	54.8 ± 2.4	54.8 ± 2.4	
Sex (%)			
Female	13 (43.4%)	13 (43.4%)	
Male	17 (56.6%)	17 (56.6%)	
Tumor diameter (cm)	6.5 ± 2.3		
Tumor stage (%)			
T1	6 (20.0%)		
T2	11 (36.7%)		
T3	8 (26.6%)		
T4	5 (16.7%)		
Market proteins (%)			
E-Cadherin (+)	7 (23.3%)	18 (60.0%)	<0.001
EZH2 (+)	21 (70.0%)	5 (16.7%)	<0.001
RUNX3 (+)	4 (13.3%)	18 (60.0%)	<0.001
β-Catenin (+)	28 (93.3%)	20 (66.6%)	>0.05

### 3.2 Construction of CCRCC cells with RUNX3 overexpression

**Figure 2:**
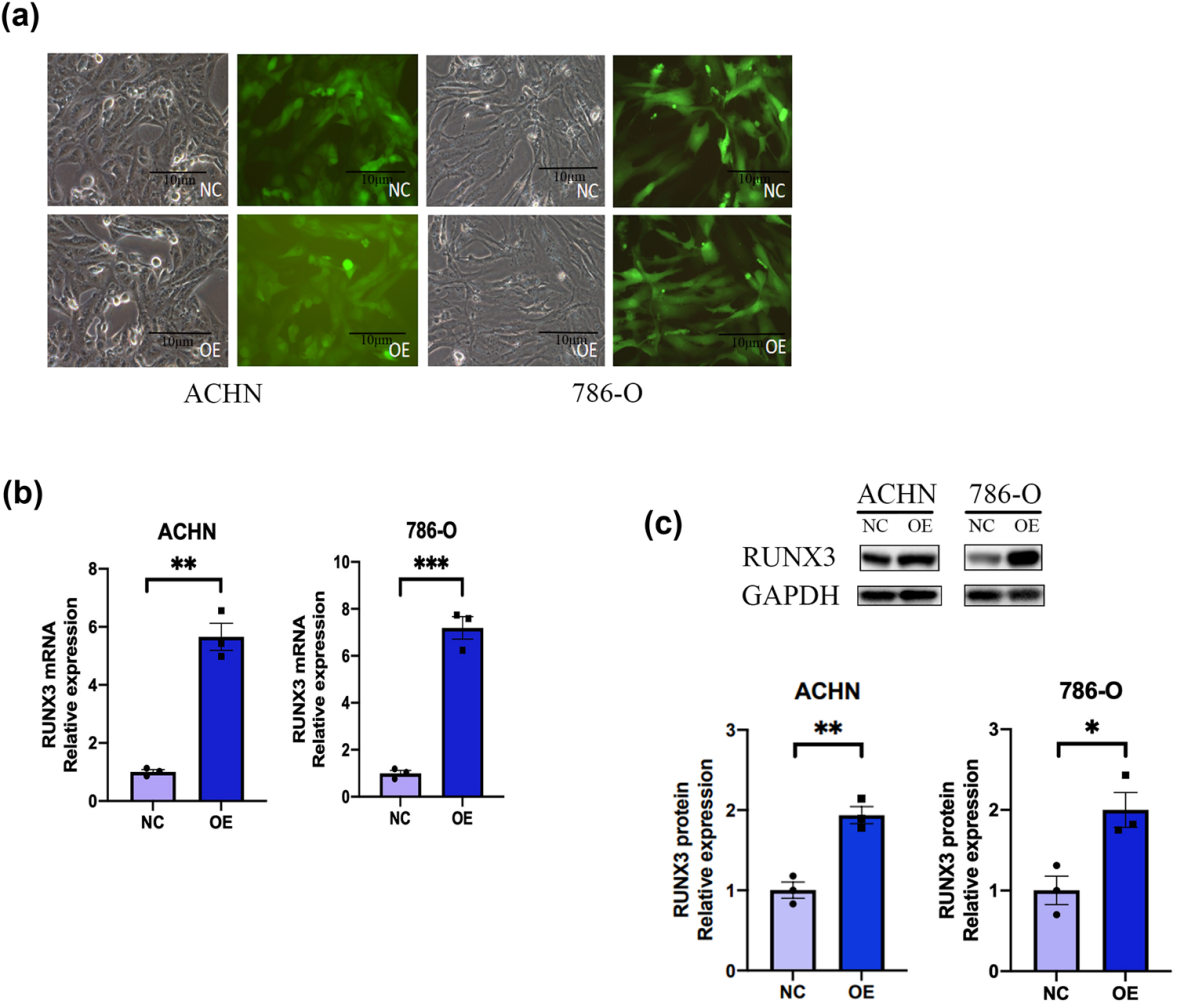
Successful transfection of two CCRCC cell lines with a *RUNX3* vector. (a) Phase contrast and fluorescence microscopy of ACHN and 786-O cell lines with *RUNX3* OE and NC cells with a control vector. GFP fluorescence of both groups indicated successful transfection (scale bar: 10 µm). (b) Expression of *RUNX3* in the two groups (RT-PCR). (c) Expression of RUNX3 in the two groups relative to GAPDH (western blotting). ImageJ was used to invert the western blot bands and measure the gray value, and data were presented as histograms. Scale bar: 10 µm, each assay was performed at least three times, and values are means ± SEMs, ***P < 0.001, **P < 0.01, *P < 0.05.

We used lentivirus transfection to construct ACHN and 786-O cells that had overexpression of *RUNX3* (OE) or NC expression of RUNX3 (NC). Fluorescence microscopy indicated successful transfection of these cells (Figure 2a). RT-PCR indicated that both types of OE cells had increased expression of *RUNX3* mRNA (Figure 2b), and we confirmed this by western blotting (Figure 2c). Thus, we successfully established CCRCC cells with overexpression of *RUNX3 in vitro*.

### 3.3 RUNX3 overexpression in CCRCC cells inhibits proliferation

**Figure 3:**
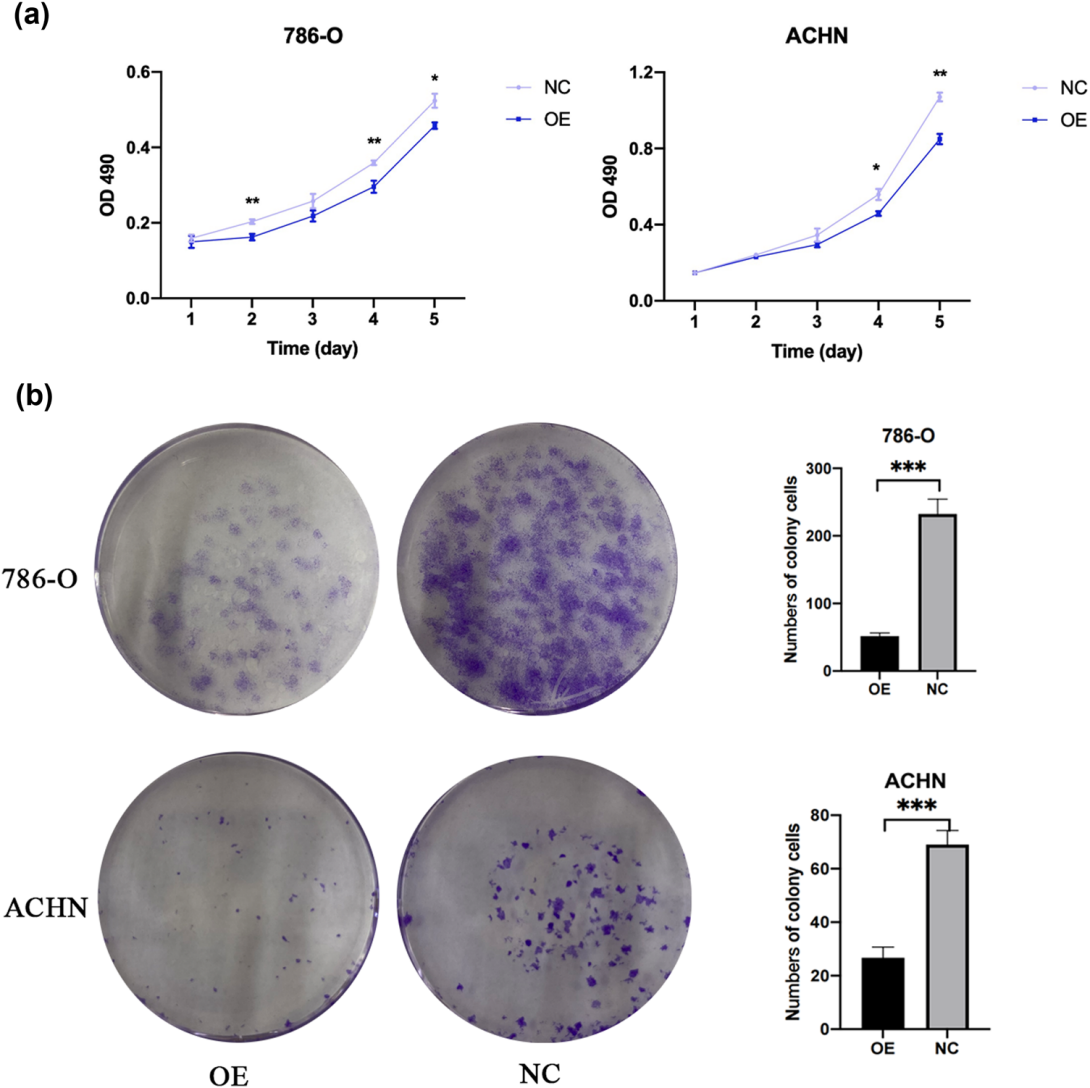
RUNX3 overexpression inhibited the proliferation of CCRCC. (a) Cell viability of the two cell lines in the OE and NC groups (MTT assay). (b) The number of cell clusters in the OE and NC groups (Cloning forming). Each assay was performed at least three times, and values are means ± SEMs, ***P < 0.001, **P < 0.01, *P < 0.05.

We measured cell proliferation of the OE and NC groups using the MTT assay (Figure 3a). For both cell lines, the cell viability of the OE group was lower than that of the NC group after 5 days. We also determined the proliferation of 786-O and ACHN cells using a clone formation assay. In both cell lines, more cell clusters formed in the OE group (Figure 3b). Thus, overexpression of *RUNX3* inhibited the proliferation of CCRCC cells *in vitro*.

### 3.4 RUNX3 overexpression in CCRCC cells inhibits invasion and migration

**Figure 4:**
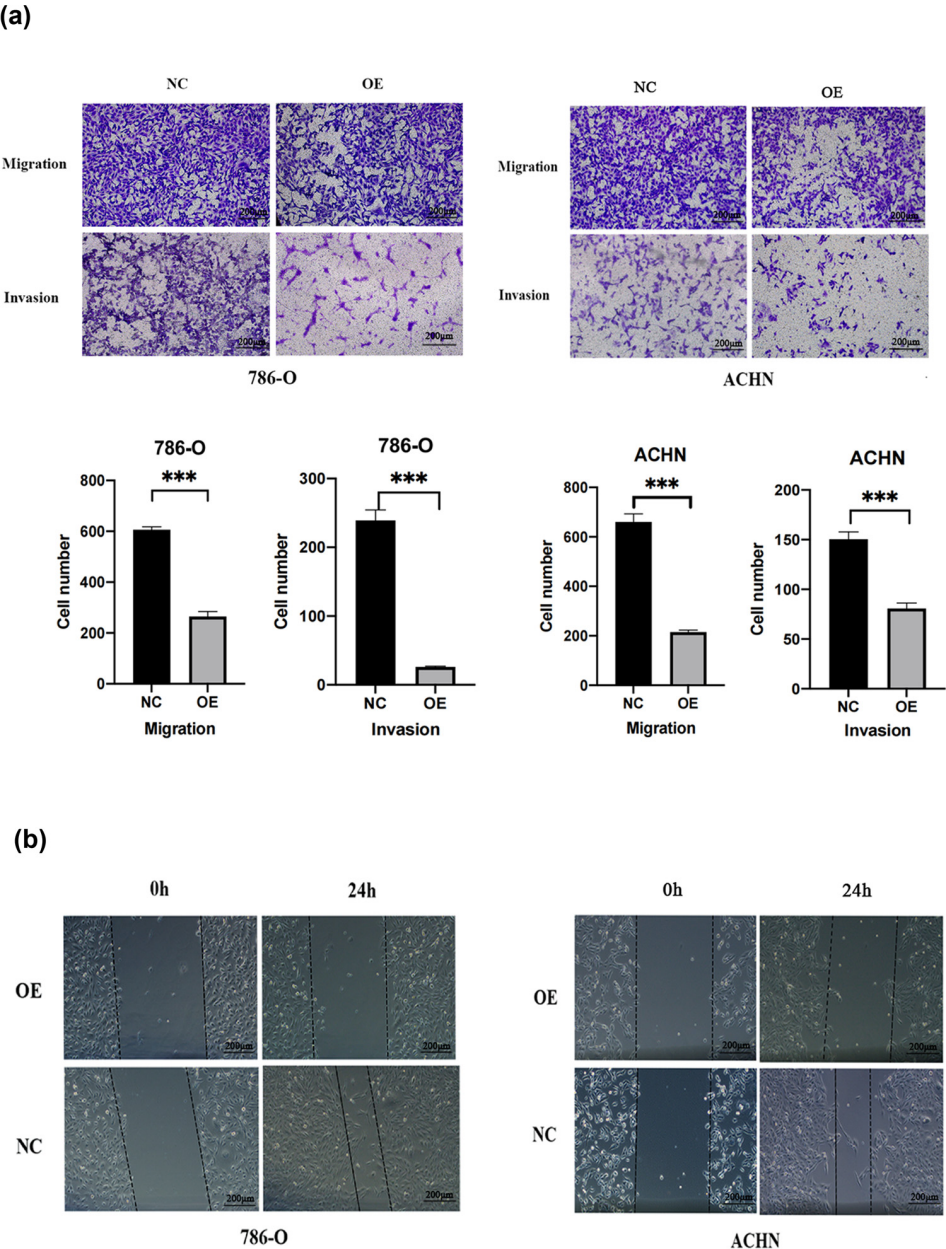
RUNX3 overexpression inhibited the migration and invasion of CCRCC. (a) Cell migration and invasion of the two cell lines in the OE and NC groups. (b) Cell migration of the two cell lines in the OE and NC groups (Cell scratch). Scale bar: 10 µm, each assay was performed at least three times, and values are means ± SEMs, ***P < 0.001.

We then examined cell invasion and migration using the Transwell assay and cell scratch assay. The results of the Transwell assay indicated that the NC groups had greater staining for migration and invasion and the OE groups had reduced cell migration and invasion (Figure 4a). The results of cell scratch assay showed that cell spacing in the OE groups did not change significantly at 0 and 24 h, but cell spacing was significantly reduced in the NC groups (Figure 4b). Thus, overexpression of RUNX3 led to reduced migration and invasion of CCRCC cells *in vitro*.

### 3.5 RUNX3 overexpression in CCRCC cells alters the cell cycle and induces apoptosis

**Figure 5:**
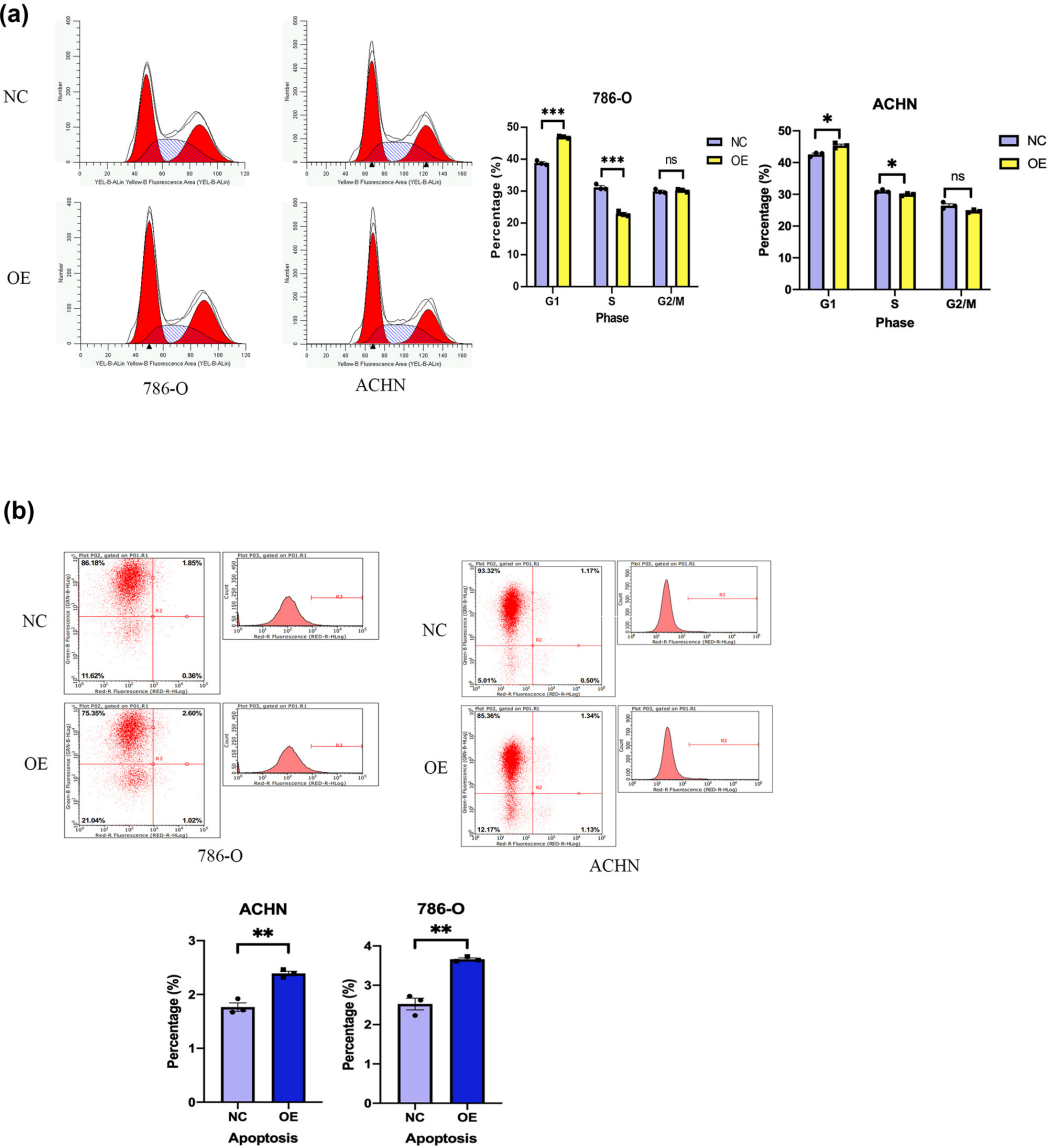
RUNX3 overexpression affects cell cycle and induces tumor cell apoptosis of CCRCC. (a) Cell cycle progression of the two cell lines in the OE and NC groups (flow cytometry). (b) Apoptosis of the two cell lines in the OE and NC groups (flow cytometry). Each assay was performed at least three times, and values are means ± SEMs, ***P < 0.001, *P < 0.05, ns *P* > 0.05.

Our cell cycle analysis of ACHN and 786-O cells indicated that the OE groups had more cells in the G1 phase and fewer cells in the S phase (Figure 5a). Our measurements of apoptosis indicated the OE groups had more apoptotic cells than the NC groups (Figure 5b). Thus, *RUNX3* disrupted the cell cycle of CCRCC cells and increased the apoptosis of CCRCC cells *in vitro*.

### 3.6 RUNX3 overexpression in CCRCC cells upregulates the expression of E-cadherin

**Figure 6:**
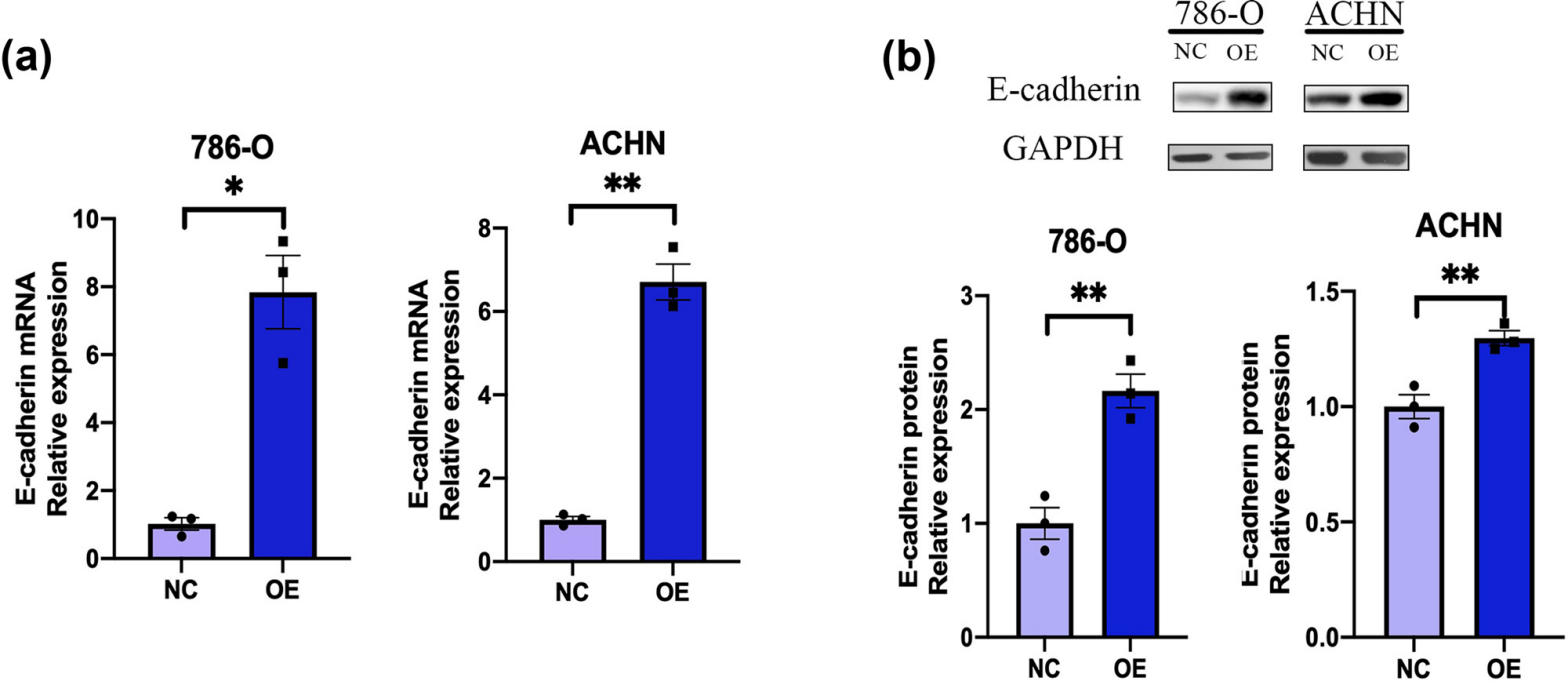
RUNX3 overexpression upregulates the expression of E-cadherin. (a) *RUNX3* expression in the OE and NC groups of the two cell lines (qPT-PCR). (b) Expression of E-cadherin in the OE and NC groups of the two cell lines (western blotting). ImageJ was used to invert the western blot bands and measure the gray value, and then the resulting data were made into histograms. Each assay was performed at least three times, and values indicate means ± SEMs, **P < 0.01, *P < 0.05.

Our qRT-PCR results showed that E-cadherin mRNA was highly expressed in the two OE groups (Figure 6a). We confirmed these results using western blotting (Figure 6b). Thus, overexpression of *RUNX3* by CCRCC cells increased the expression of E-cadherin mRNA.

### 3.7 Cells with the OE vector have decreased tumorigenicity in mice

**Figure 7:**
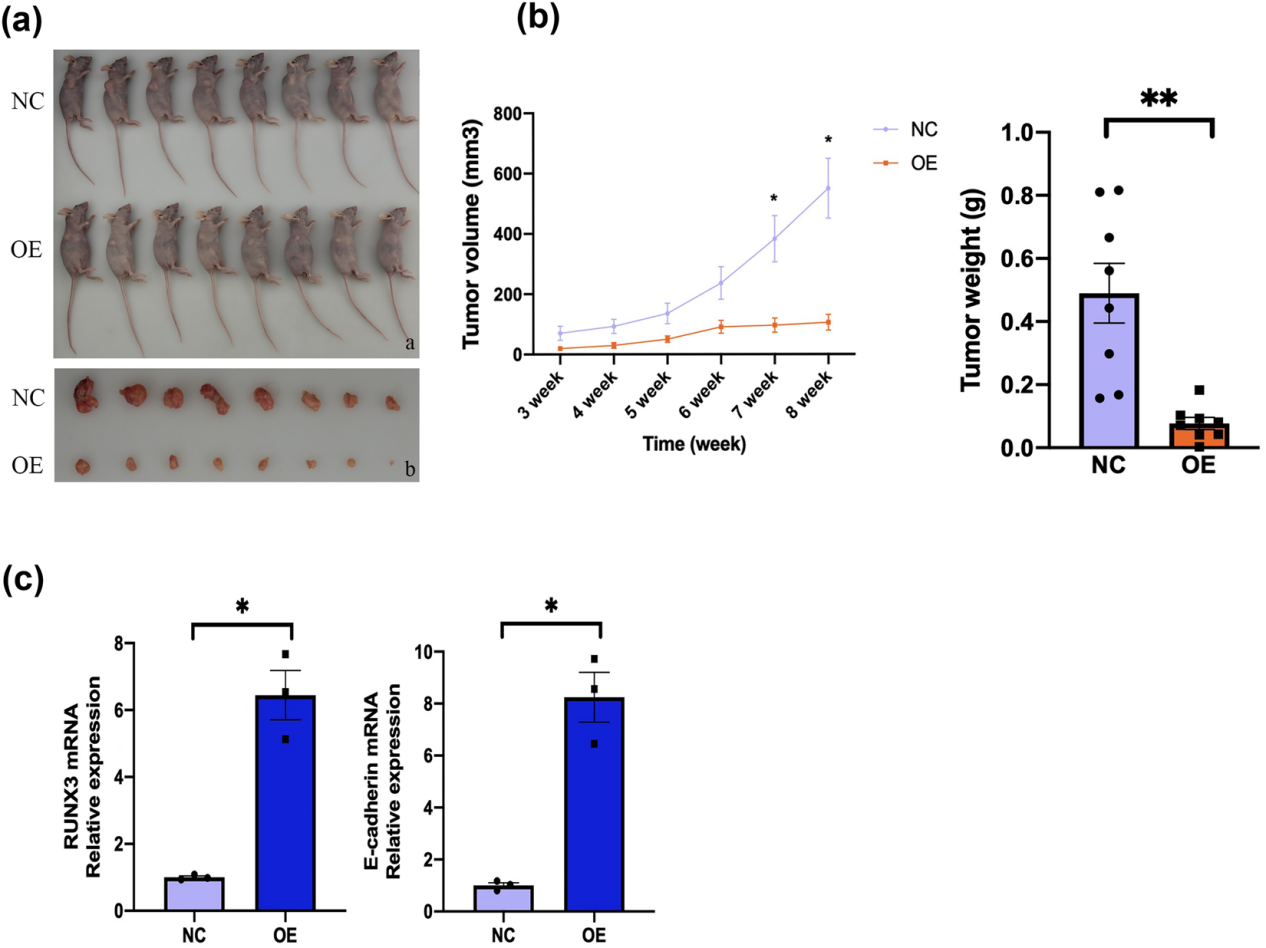
Cells with the OE vector have decreased tumorigenicity in mice. (a) Mice and tumors after injection of tumor cells in the OE group (top rows) and NC group (bottom rows). (b) Tumor volume and weight of mice in the OE and NC groups. Calipers were used to measure the length (*L*), width (*W*), and height (*H*) of the tumor tissue, and volume (*V*) was then calculated: *V* = π/6 × *L* × *W* × *H*. (c) Expression of *RUNX3* and E-cadherin in mouse tumor tissues after 56 days (qRT-PCR). Each assay was performed at least three times, and values are means ± SEMs, ***P < 0.001, **P < 0.01, *P < 0.05.

We then performed *in vivo* experiments in which nude mice received subcutaneous injections of ACHN cells that were transfected with the *RUNX3* OE vector or the NC vector. After 6 weeks, the tumor volume and tumor weight of mice that received NC cells were significantly greater than those of mice that received OE cells (Figure 7a and b). Measurements of the expression of *RUNX3* and E-cadherin mRNAs in mouse tumor tissues also indicated greater expression of these genes in mice that received the OE cells (Figure 7c). Thus, *in vivo* overexpression of *RUNX3* in CCRCC xenografts led to reduced tumor volume and weight and increased expression of E-cadherin.

## 4 Discussion

CCRCC is the most common pathological type of RCC and it also has a high rate of recurrence. In particular, Dabestani et al. studied European patients with CCRCC, and found that 286 of 1,265 of them had recurrence [21]. Therefore, further research is needed to examine possible causes of proliferation, metastasis, and recurrence of CCRCC. For example, new biomarkers that can be used to monitor tumor progression or as drug targets may be particularly useful. The EMT is an important biological process that functions in fibrosis and tumor metastasis. At the cellular level, the EMT is associated with enhanced cell migration and cell invasion, reduced apoptosis, and increased production of extracellular matrix [22,23]. E-cadherin has a key function in the EMT, and a reduced expression of this protein is related reduced cell adhesion and increased metastasis [24].

In this study, we first examined the expression of *RUNX3* in cancer tissues and adjacent normal tissues of 30 patients with CCRCC. Our results indicated that *RUNX3* expression was significantly lower in CCRCC tissues than in adjacent normal tissues of the same patient. This result is consistent with studies of other malignant cancers and indicates that *RUNX3* functions as a tumor suppressor gene in CCRCC [25,26]. We also measured the expression of E-cadherin, β-catenin, and *EZH2*. Our results showed that the expression of E-cadherin was lower in CCRCC tissues than in non-cancerous tissues. EZH2 is a highly expressed protein in tumor tissues and promotes tumor formation by downregulating E-cadherin [27]. In agreement, we also found overexpression of *EZH2* in CCRCC. To examine the specific function of *RUNX3* and its role in the EMT process during the progression of CCRCC, we further examined RUNX3 and E-cadherin as target proteins.

Thus, we validated the proliferation of CCRCC cells using the MTT and clone formation assays. The results indicated that two lines of CCRCC cells that overexpressed *RUNX3* had lower proliferation. Previous studies demonstrated that *RUNX3* overexpression inhibited the proliferation of non-small-cell lung cancer cells [28], consistent with our results. Metastasis is a tumor-specific biological behavior, and cell migration and invasion are critical for this process. The results of our cell scratch assay and Transwell assays on 786-O and ACHN cells showed that cell lines with the overexpression of *RUNX3* had enhanced cell invasion and migration. Thus, our findings demonstrated that *RUNX3* inhibited the invasion and migration of CCRCC, similar to its function in other cancers.

Our examination of the effect of *RUNX3* on the cell cycle and apoptosis of CCRCC cells indicated that cells with overexpression of *RUNX3* had an accumulation of cells in the G1 phase, and a fewer cells in the S phase. These results indicated that overexpression of *RUNX3* prolonged the cell cycle of CCRCC cells. We also measured apoptosis using Annexin V-APC single staining and TUNEL staining. In agreement, these results showed there was increased apoptosis in cells that had overexpression of *RUNX3*. As above, these results are also consistent with previous research that examined the function of *RUNX3* in ovarian cancer, gastric cancer, colon cancer, and other cancers [29,30,31].

Our measurements of the expression of E-cadherin in two lines of CCRCC cells (786-O and ACHN) showed that this protein had greater expression when *RUNX3* was overexpressed. E-cadherin has an essential function during the EMT, and the EMT plays an important role in tumor invasion and metastasis. Our *in vitro* results thus lead to the conclusion that *RUNX3* functions in the EMT by promoting the expression of E-cadherin. We also examined the function of *RUNX3* by performing *in vivo* experiments using a nude mouse xenograft model of CCRCC. The results showed that mice that received CCRCC cells with *RUNX3* overexpression had a slower rate of tumor formation and reduced tumor size and weight. Our measurements of the expression of E-cadherin in mouse tumor tissue confirmed that E-cadherin expression and *RUNX3* expression were positively correlated. These results, which are consistent with our *in vitro* experiments, demonstrated that *RUNX3* functioned in the EMT by upregulating E-cadherin.

In conclusion, our results confirmed that *RUNX3* functioned as a tumor suppressor gene in CCRCC. More specifically, *RUNX3* functions as a tumor suppressor by upregulating the expression of E-cadherin, a protein that has a key function in the EMT. We therefore believe that *RUNX3* expression may be useful for predicting the recurrence or metastasis of this tumor, and it also has potential use as a novel target in future studies that examine drug therapies for CCRCC.
